# Fiber Optic Sensor Embedment Study for Multi-Parameter Strain Sensing

**DOI:** 10.3390/s17040667

**Published:** 2017-03-23

**Authors:** Monssef Drissi-Habti, Venkadesh Raman, Aghiad Khadour, Safiullah Timorian

**Affiliations:** 1 PRES LUNAM IFSTTAR CS4 Route de Bouaye, 44344 Bouguenais, France; safiullah.timorian@ifsttar.fr; 2 Institut de Recherche Technologique (IRT) Jules VERNE, Chemin du Chaffault, 44340 Bouguenais, France; raman.venkadesh@irt-jules-verne.fr; 3 Components and Systems Department, Université Paris-Est, IFSTTAR, 77420 Champs-sur-Marne, France; aghiad.khadour@ifsttar.fr

**Keywords:** structural health monitoring (SHM), fiber optic sensors, composite material, bonding, numerical simulation, finite element analysis, distributed sensors, multi-axial, multi-parameter strain

## Abstract

The fiber optic sensors (FOSs) are commonly used for large-scale structure monitoring systems for their small size, noise free and low electrical risk characteristics. Embedded fiber optic sensors (FOSs) lead to micro-damage in composite structures. This damage generation threshold is based on the coating material of the FOSs and their diameter. In addition, embedded FOSs are aligned parallel to reinforcement fibers to avoid micro-damage creation. This linear positioning of distributed FOS fails to provide all strain parameters. We suggest novel sinusoidal sensor positioning to overcome this issue. This method tends to provide multi-parameter strains in a large surface area. The effectiveness of sinusoidal FOS positioning over linear FOS positioning is studied under both numerical and experimental methods. This study proves the advantages of the sinusoidal positioning method for FOS in composite material’s bonding.

## 1. Introduction

The structural maintenance of large civil, aeronautic and wind turbine structures is difficult to establish with periodic verification methods. Maintenance works of these structures are conducted by non-destructive testing (NDT) most of the time [1]. Material development in these sectors demands high technology equipment and qualified man power, which increase the maintenance process cost. The real-time monitoring method provides the structural behavior and eases the maintenance work. This method is called structural health monitoring (SHM). SHM can alert the operators to anomalies and failures. Online, continuous and global SHM can reduce maintenance and component replacement cost by monitoring structures under harsh environments in remote sites. SHM offers robust real-time monitoring, because necessary maintenance or repairs could be addressed based on this technology [1]. Life fatigue and economic issues are arising in industrial composite structures with age. SHM could offer a vital tool to inspect continuously for potential failures in the systems.

In SHM, the sensors are the fundamental compound to provide information about the performance of the structure. Important procedures in SHM are the selection and placement of suitable sensors for measuring key parameters. There are several parameters to monitor in large structures, like: pressure, temperature, strain and humidity. The strain sensors provide more information about structure and material properties [2]. In multiple commercial applications, FOSs have been already selected for health monitoring. The greatest advantage of FOSs is their intrinsic link to other optical fibers. The links allow the sensor to keep the quality of the signal with low loss. Therefore, the sensor functions can extend for long distances. In almost all FOS applications, the optical fiber is a thin glass fiber that is protected mechanically with a polymer coating and inserted in a cable [3]. The sensors can be placed on the surface of the structure or embedded within the structure.

The light signals travel inside the optical fiber. The transmitted and reflected light is modulated by its amplitude, phase, frequency or polarization state. If the structure undergoes the strain effect, there is a change in light transmission parameters, which is converted to the strain rate applied to the structure [4]. The most commonly-used sensors are grating based (FBG (Fiber Bragg grating)) and distributed sensors. The distributed sensor works by the back-scatter method. The strain can be measured by the time-domain technique (OTDR) or the frequency-domain technique (OFDR). Sensing solutions based on back-scatter make the use of off-the-shelf low cost telecom-grade fiber as a sensor possible.

FOS is very small in size and flexible. The addition of polymers around the glass core provides additional flexibility to FOS. This characteristic allows the optical fibers to be inserted successfully inside the composite structures. The perturbation of the surrounding field in the host due to the presence of the sensor is often termed the ‘obtrusivity’ of the sensor. Excessive obtrusivity will not only perturb the values of the field variables being measured, but affect the integrity of the host, sensor and the interface between them. The resulting degradation can compromise the accuracy of measurements and the long-term reliability of the sensor-host interaction [5]. While embedding FOS between the composite plies, it creates a resin concentration called ‘resin pockets’ that disturbs the obtrusivity of the sensors. This will modify the stress transfer between composite plies and FOS. The sensors are positioned in the composite material parallel to the composite fiber alignment directions to reduce the dimension of the resin pockets [6]. However, laminate orientations may vary to reinforce the composite structure. Accordingly, the resin concentration is more than the expected level, and that affects the stress transfer between the FOS and composite material [7]. The possible embedded optical fibers to use in anisotropic materials have already been demonstrated [8,9]. There are two main coating materials used in industrial applications without considering outer buffer coats: acrylate and polyimide. They both have different mechanical properties and cost effects. Polyimide has a higher modulus than the acrylate, so it withstands high mechanical loads and harsh environments like salt and temperature [10]. On the contrary, the cost of the polyimide is higher than acrylate; therefore, it is difficult to choose a suitable solution for large-scale structures.

In this paper, we will discuss the stress transfer between the coating material and epoxy material. We consider the epoxy material as a bonding between two composite thin parts in order to make the proper stress analysis about the coating influence. Several previous studies do not investigate in detail the out-of-plane strain, which is a key parameter of structural integrity in anisotropic materials. Castellucci et al. [11] studied the out-of-plane sensitivity in a 3D-woven cloth configuration with distributed strain sensors. Their study is concentrated on sensor spacing and gauge length to collect the strain all the way through the FOS. In recent years, substantial research efforts also attempted developing multi-parameter FBG sensors [12,13]. They worked on multi-axial strain observation in grating sensors. A change in axial strain or temperature will not affect the birefringence of the fiber, but that will cause an overall shift of the Bragg spectrum towards higher or lower values. A change in transverse strain leads to a change of the birefringence of the fiber, which results in a change of the peak separation. Therefore, they proposed capillary configuration for the Bragg grating sensor obtains axial and transversal strain. The sensitivity to transversal strain also remained considerably lower than the sensitivity to axial strain in that solution. Several reports described the characterization of gratings subjected to transversal strains [14,15]. Disbond area and crack prediction under fatigue loadings are also possible by FBG sensors [16,17]. Changing the sensitivity property of coating and cladding also allows the multi-parameter strains’, physical and chemical properties’ (gas, humidity) detection in grating sensors [18,19,20]. We are using the distributed FOS in our study; the multi-axial strains can be identified by the optimal positioning of the sensors in the composites. This alignment of FOS is assumed as the sinusoidal form. Therefore, we make a numerical study of the embedded sensor [21] in the composite material bonding. The same sinusoidal fiber alignment model is difficult to design with the resin pocket effect. Fibers have their own limit of the bending angle to avoid fiber breakage [22]. The optimal sinusoidal profile should be selected in the simulation. In this study, we will see the strain detection in different directions by various optical fiber alignment methods. As two coating materials are considered in this work, their presence was taken into account during stress transfer analysis. Once the multi-axial strains are proven by the numerical model, the experiments were established for the alignment method study. The distributed FOS is bonded over the composite specimens by epoxy bonding material to check the effectiveness of the fiber optic alignment.

## 2. Objectives

**Figure 1:**
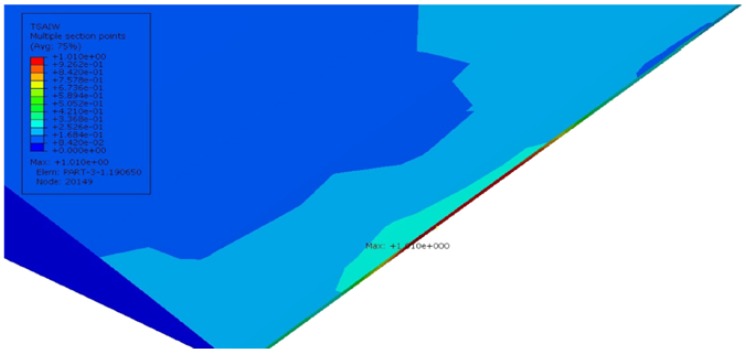
Blade bonding damage observed in the numerical study, source: [24].

**Figure 2:**
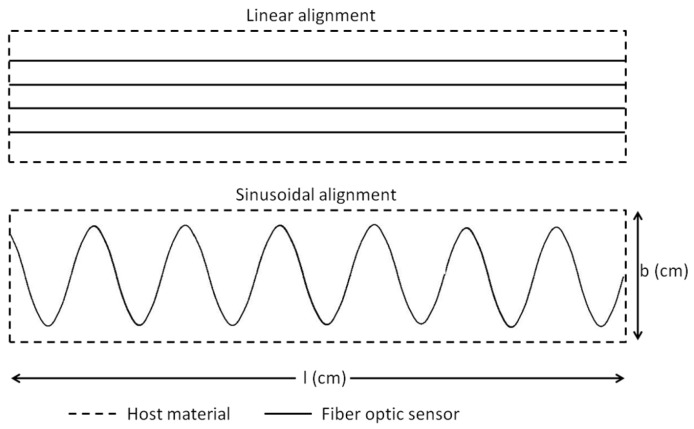
Fiber optic sensor (FOS) installation method for a reference surface area.

Composite wind turbine blades are made of two separate sections and bonded by suitable adhesive materials [23]; see Figure 1. This adhesive bonded area is more sensitive to damage, as this is the fragile compound of the blade [24]. In order to achieve the SHM process, these zones should be “watched” properly. Two main options of the placements of optical fiber sensors between the bond joints are therefore considered (see Figure 2). The first one is the embedment of straight parallel optical fibers, whereas the second is based on the embedment of a sinusoidal optical fiber. As the bonding zones are wider and the stress-strain field is random, monitoring needs multiple placements of straight optical fibers, which will be costly (the need for many costly optical back-scattering (OBR) facilities). The alternative option of placement that we are suggesting is based on the embedment of a single sinusoidal optical fiber with appropriate periodicity that may enable the monitoring of the entire joint. In addition, the strain values close to the edge are studied for the edge effect in order to avoid the fiber slippage and sensibility reduction. Our study provides the advantages of the sinusoidal positioning method of the FOS installation for large-scale application. This study gives an overview and solution of FOS installation to industrial wind turbine blade bonding SHM designers.

## 3. Numerical Simulation

### 3.1. Numerical Modeling

The alignment method of FOS is studied in the composite material bonding zone by the numerical method. The FOS is considered with both acrylate and polyimide coating materials as they are used generally for industrial application. Our study focuses on providing a clear overview about the fiber alignment. There are some finite element analyses that are realized for the mechanical and thermal stress study between FOS and host materials [25,26,27]. The model is developed using Abaqus commercial software. The numerical model of the specimen is optimized with a dimension of (10 × 2 × 0.66) mm for the finite element analysis. It is made of three main parts, including optical fiber sub-parts. The FOSs are designed with the significant coating material properties mentioned in Table 1. Two carbon fiber composites are bonded together with epoxy matrix, which hosts a linear and sinusoidal, aligned optical fiber.

At the end, the most efficient and cost-effective embedded alignment technique will be proposed for industrial applications. The linear model and sinusoidal alignment models were modeled in the same way, but the sensor alignment makes the model very complicated for the sinusoidal compared to the linear model. The outer cuboid geometry shapes represent the composite materials with a 0.25-mm thickness. As this study is for analyzing the strain variation around the fiber optics, the dimensions are considered to visualize the strain around the FOS with low meshes in order to lower the simulation period. Design: In the rectangular model, the centered rectangular section is considered as the epoxy bonding material with a 0.16-mm thickness. The circular section in the epoxy model represents the embedded FOS, including the coating, cladding and core. The coating is modeled with acrylate and polyimide for the comparative analysis; the cladding and core were designed with the silica glass material property. The diameter of the FOS is considered as 0.125 mm and positioned in the middle of the epoxy. In the linear FOS alignment model, the fiber is aligned along the lengthwise direction of the specimen. The material properties are mentioned in Table 1.

**Figure 3:**
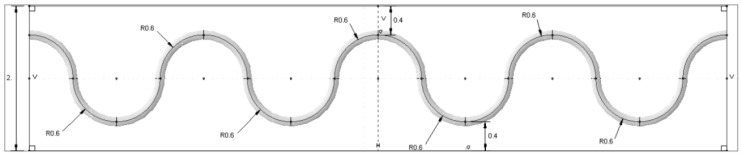
Sinusoidal method FOS alignment; design factors with dimensions.

**Figure 4:**
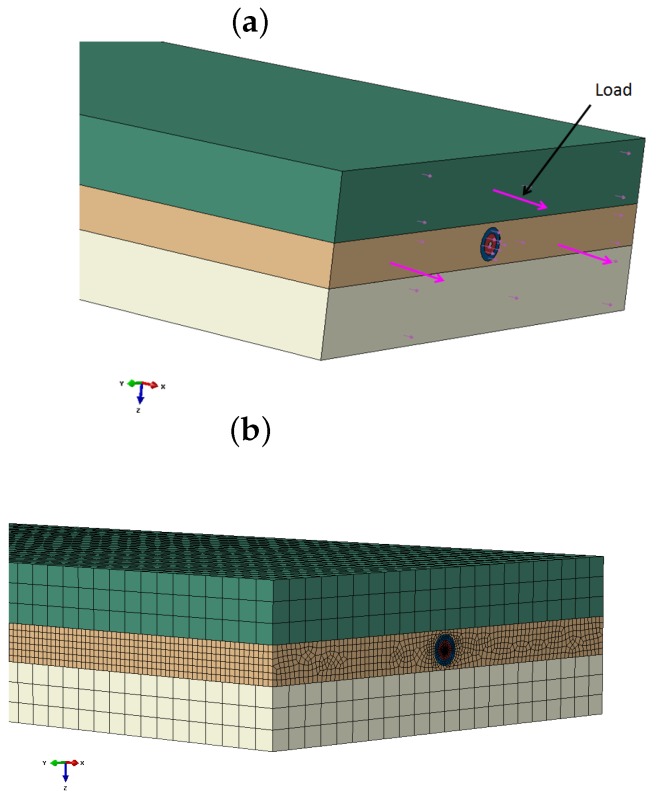
(**a**) Surface traction load applied in the numerical model (the rose arrow indicates the direction of the load applied); (**b**) numerical specimen model with mesh.

**Figure 5:**
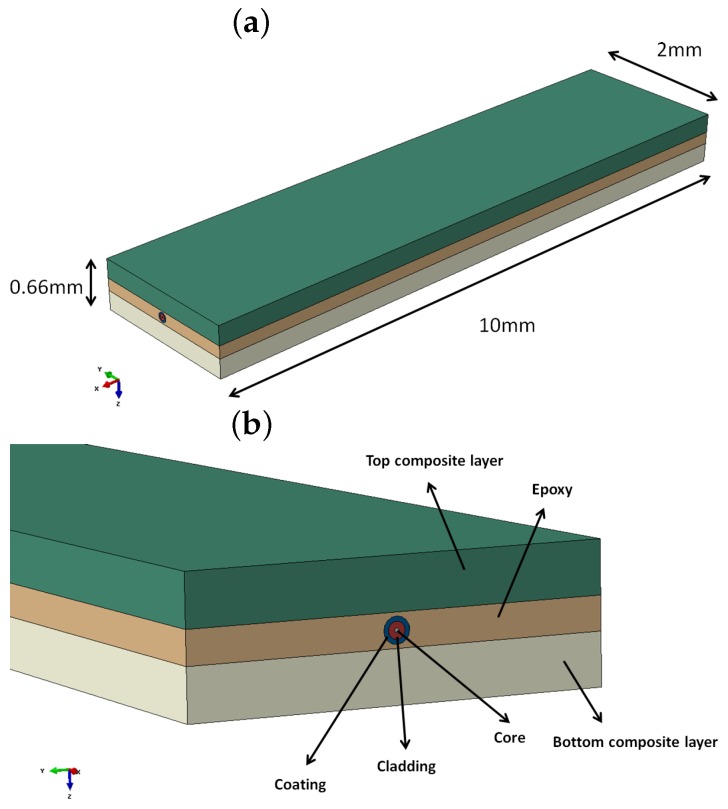
(**a**) Fiber optic sensor embedded in adhesive bonding in between composite plies: numerical model geometry; (**b**) numerical specimen model described with components.

For the sinusoidal model, also the FOS is aligned towards length-wise of the specimen with the sinusoidal peaks as shown in Figure 3. Mesh: The finite element analysis over this specimen is considered under different mesh conditions. All parts of the specimen are meshed with different global seeds to fulfill the most advanced numerical concentration around the focal point of analysis. As the specimen model is created with different material properties, one should take into account the isotropic and composite material property during analysis. The first upper and lower layers of the model are composite laminate with a 0${}^{\circ }$ orientation. As for the complexity of the geometry, model is meshed with structured, free tetrahedral and boundary optimized free tetrahedral. The mesh size is varying for each part of the model; see Figure 4b. The fine mesh is used around the FOS to avoid calculation error and distortion. Boundary condition: In this model, the load is applied as the tensile load with 400 N in each specimen’s outer side axial edges; see Figure 4a. The load is applied in a surface traction manner. The displacement is allowed in the X-direction. The rotation is blocked in the Y- and Z-directions. The coordinate system of all of the parts is oriented with the global coordinates; see Figure 5a.

The numerical model is submitted to quasi-static loading with the boundary conditions mentioned above.

### 3.2. Factors Analyzed in the Numerical Model

**Figure 6:**
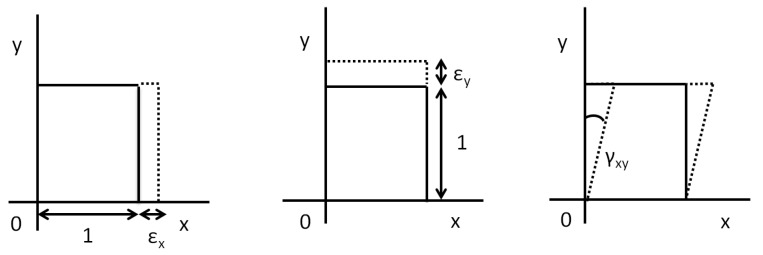
Strains defined based on coordinates in the plane-strain model.

The result of the numerical model is analyzed based on multi-directional strains; they are linear, lateral and shear strains. This can be explained by the plane-strain model. The coordinate is considered OX-OY-OZ, as X-Y-Z [28]. The lateral strains are equal in the direction of Y and the direction of Z, because we are using the orthotropic assumption. Accordingly, the strain components are explained in the X-Y plane. Assume a plane strain model (see Figure 6) with ‘l’ length (OX-direction), and the bar is subjected to the uniaxial tensile load along the length direction. The load is applied with the ‘F’ (N) value (OX direction). After the load, the element extends with ‘$\delta_{l}$’ (mm) variation. Linear strain of a deformed element is defined as the ratio of the change in length of the element due to the deformation to its original length in the direction of the force. The linear strain ‘$\epsilon_{x}$’ (mm/mm) is given by $\epsilon_{x}$ = $\delta_{l}$/1 = $\delta_{l}$. The linear strain could be tensile or compression strain according to the direction of the element length variation. In our case, the tensile linear strain is considered. Lateral strain $\epsilon_{y}$ is the ratio of the change in length (breadth of a rectangular bar or diameter of a circular bar, OY-direction) of the body due to the deformation to its original length in the direction perpendicular to the force. In addition, load acting on the surface of the model creates the sliding effect in the cross-section (OX parallel direction). This sliding stress creates the section variation with an angle to the normal direction (OY-direction); this angle represent the shear strain ($\gamma_{xy}$). In our simulation, we are considering these strain components to analyze the multi-strain sensing characteristics of sensor alignment as follows, $\epsilon_{x}$ = $E_{11}$, $\epsilon_{y}$ = $E_{22}$ and $\gamma_{xy}$ = $E_{12}$.

## 4. Experimental Preparation

**Figure 7:**
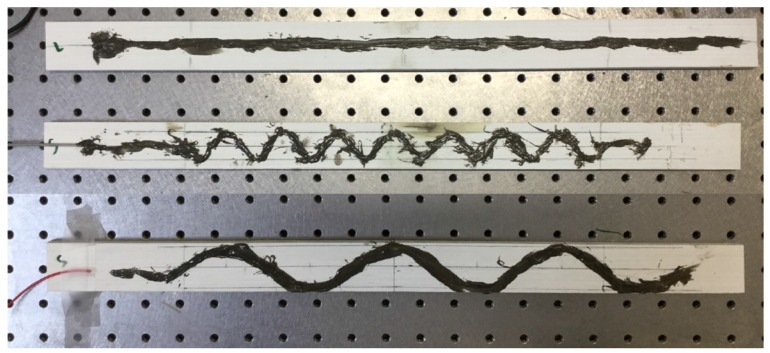
Method of FOS alignment in the bonding zone for the experimental model; linear, small sinusoidal and extended sinusoidal alignments represented from top to bottom.

The effectiveness of FOS alignment is also tested in mechanical test under three-point bending load. The specimen was prepared with bonded FOS. The glass fiber-reinforced composite material made by the pultrusion method is used for the experiment. The specimen was cut in the dimensions of (500 × 35 × 7) mm. The FOS with a 0.08-mm cladding diameter surrounded by a 0.125-mm diameter acrylate coating is bonded over the composite material specimen. The bi-component epoxy resin is used for the bonding. The FOS is bonded as shown in Figure 7 with equal intervals. The peak is maintained at the middle of the specimen and spread towards the tip of the specimen linearly. Two different diameters are considered for the sinusoidal form; they have 2-cm period waves (small sinusoidal alignment) and 6-cm period waves (extended sinusoidal alignment). This period change is collected from edge effect study of the numerical simulation that will be mentioned in Section 5.1. For the linear model, the optical fiber is positioned exactly at the middle (width wise) of the specimen. To maintain the optical fiber’s position, they are bonded initially on the work bench, as well as the composite specimens. This helps to avoid the fiber fault and misalignment during the bonding. Once the bonding is realized with a small wooden stick at ambient temperature, they were sent to an industrial ovenunder 40${}^{\circ }$C temperatures as recommended by the adhesive’s producer. After one day of being completely placed in the oven, the specimen was checked for the state of fiber bonding. Afterwards, another composite plate was kept over this and sent to mechanical experiments. Once again, the quality of the specimen is checked; the fiber optic connectors are linked with the FOS. For the mechanical loading, the specimen was subjected to an imposed displacement controlled load at the middle of the specimen to get the bending behavior. The torsion is applied on the specimen by the help of the wedge support. The wedge angle provides the quantity of torsion displacement. The imposed displacement is 3.5 mm, and the wedge angle is maintained as 45${}^{\circ }$.

The distributed FOS is treated with LUNA OBR-4600 type optical back-scatter reflectometry equipment. The OFDR is used with the Rayleigh scatter signal in a single mode fiber. We have considered a distributed Rayleigh sensor for both the numerical and experimental study. The strain value is measured with the continuous monitoring method. The sensor was functioning with a 1550-nm wavelength, and the frequency of strain acquisition is 1 Hz. The equipment spatial length is 0.5 cm.

## 5. Results

### 5.1. Results of Numerical Simulation

**Figure 8:**
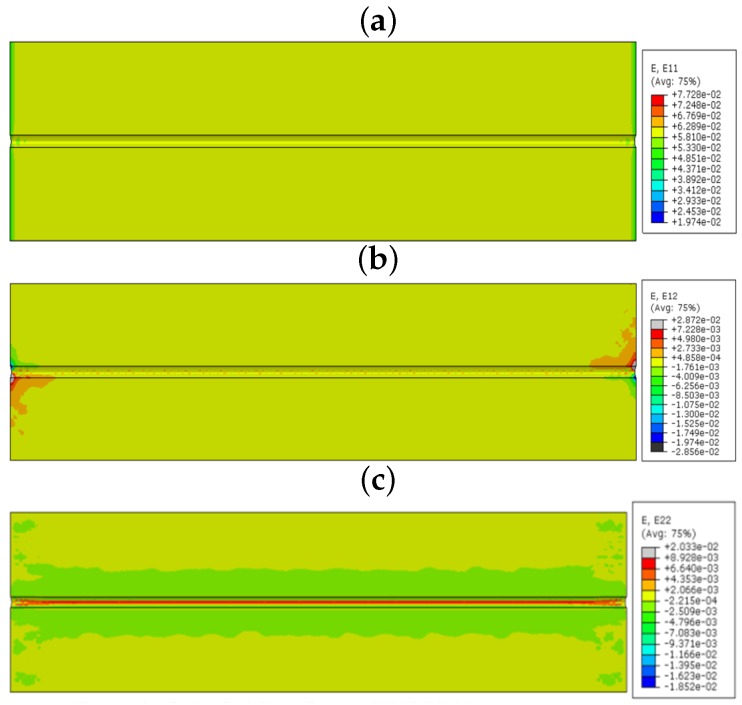
(**a**) $E_{11}$ strain component; (**b**) $E_{12}$ strain component; (**c**) $E_{22}$ strain component (acrylate-coated FOS numerical model; the value observed in the host epoxy) linear alignment.

The model is simulated using the static loading condition with the standard increment proposed by the Abaqus software. The results were mainly concentrated in epoxy, which surrounds the FOS, because the stress concentration is high in this area. Initially, the model is simulated with acrylate fiber optic coating. Figure 8 shows the linear, shear and lateral strains in the bonding area. The unit of strain in the numerical part is mm/mm. In the longitudinal direction ($E_{11}$), there is no strain observed in the interesting zone, as the maximum strain reaches 0.772 at the loading point. Shear strain inside the embedded space of the coating is $E_{12}$, which has effects only on the left lower corner and right upper corner of the part. The red spots seem to have the max value of 0.0287. Figure 8 also shows the strain effects in the lateral direction ($E_{22}$) with the contour color representation of red in the interaction surface between the epoxy and coating of the optical fiber. The maximum value of strain is 0.020331, which is higher than the shear and longitudinal strain in the zone of interest. The epoxy undergoes very low linear and shear strains, because the variation of length in FOS and bonding material is similar. Therefore, no strain was observed in the longitudinal direction. The shear is observed as very low, as the load is applied towards the direction of the fiber. The shear strain has some effect on the edge of the specimen, because of the load applying manner. The zone of interest feels small shear strain, but the stress transfer to the FOS is low in this section. The lateral strain concentration clearly takes place in the epoxy region, and it is visible, as the stress transfer is high in the lateral direction.

**Figure 9:**
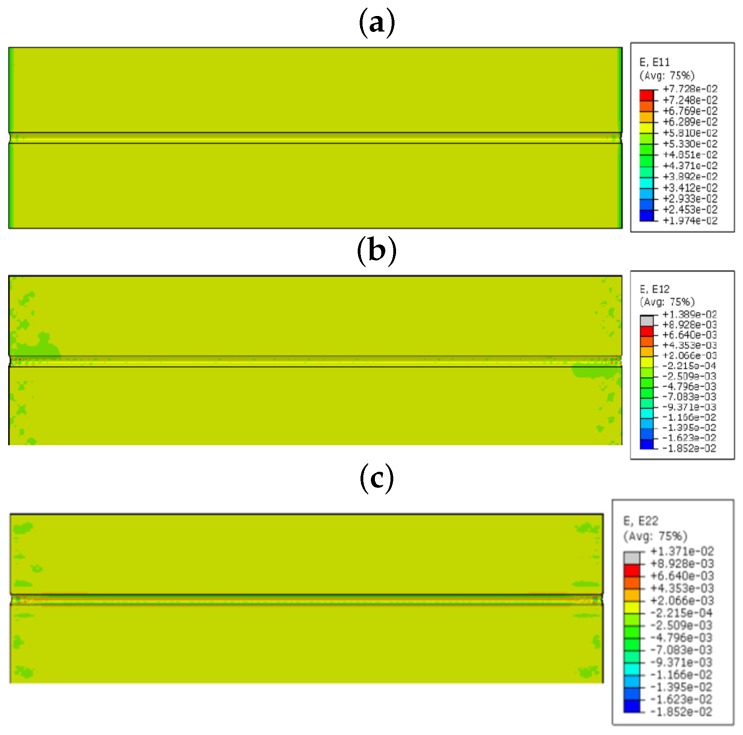
(**a**) $E_{11}$ strain component; (**b**) $E_{12}$ strain component; (**c**) $E_{22}$ strain component (polyimide-coated FOS numerical model; the value observed in the host epoxy) linear alignment.

Afterwards, the model is simulated with polyimide fiber optic coating material. The result with the polyimide-coated fiber optics seems to be very similar to the acrylate-coated FOS model. As the focal point of interest is the strain measurement beyond the interaction surfaces of the epoxy and polyamide coating, the analysis is concentrated inside the epoxy adhesive, as it has a very sensitive behavior. Figure 9 shows the longitudinal strain effects in the epoxy adhesive, and the interesting part is that it has completely the same behavior as acrylate. The deformed model in the longitudinal direction ($E_{11}$) has the same contour and strain values all over the part; there is no strained zone observed, and the maximum strain reaches 0.0772. The maximum shear strain over the part reads 0.01389, which is much less than the acrylate coating with the max value of 0.0287. Figure 9 also shows strain effects on the lateral direction ($E_{22}$) with a reduced contour color concentration compared to the acrylate model, and it represents less stress transfer, because polyimide is more rigid. The red spot in the interaction surface between the epoxy and coating of the optical fiber with the maximum value of strain is 0.01371, which is lower than acrylate, as well as the shear and longitudinal strain in the polyamide model.

**Figure 10:**
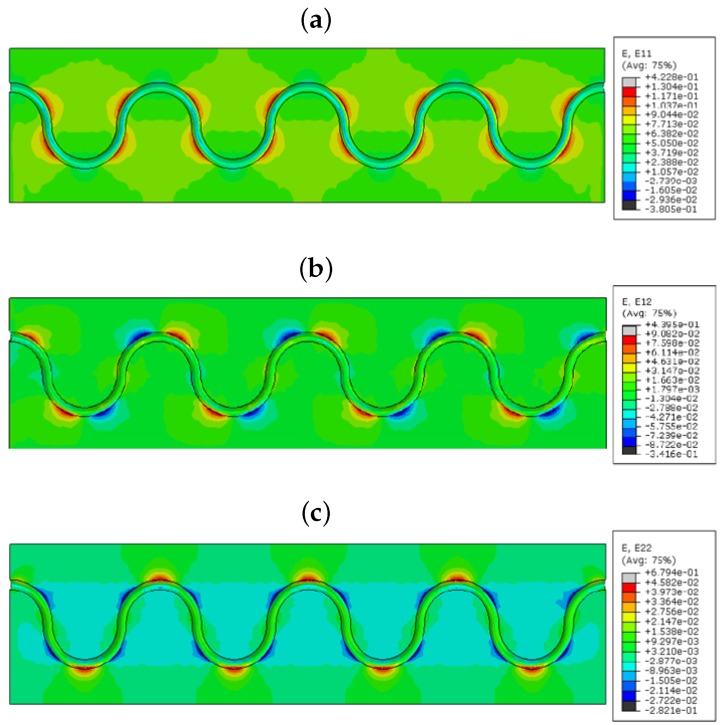
(**a**) $E_{11}$ strain component; (**b**) $E_{12}$ strain component; (**c**) $E_{22}$ strain component (acrylate-coated FOS numerical model; the value observed in the host epoxy), sinusoidal alignment.

The study of sinusoidal aligned fiber is done under the same boundary condition. Figure 10 shows the linear, lateral and shear strains, respectively, for a model with acrylate-coated FOS aligned in a sinusoidal method. Figure 10a demonstrates the behavior of the strain effects in the longitudinal direction of the numerical model with fiber optic acrylate coating. It can be seen that the red topologies in the quarter degrees of the curvatures have great tension (red), and this allows the coating of the optical fiber to force the epoxy at those regions and predicts the strains on those parts based on applying the forces. The maximum strain value over that spot reads 0.422, and it gradually decreases on the mid-horizontal axis of the structure. This trend continues till the end of the numerical model with the symmetrical shapes and expresses higher strain values on other side of fiber regions. The minimum strain appears in the peak of the sinusoidal curve with the value of 0.283. Ultimately, strain in the longitudinal axis creates high a stress concentration, which is shown in the red color contour.

**Figure 11:**
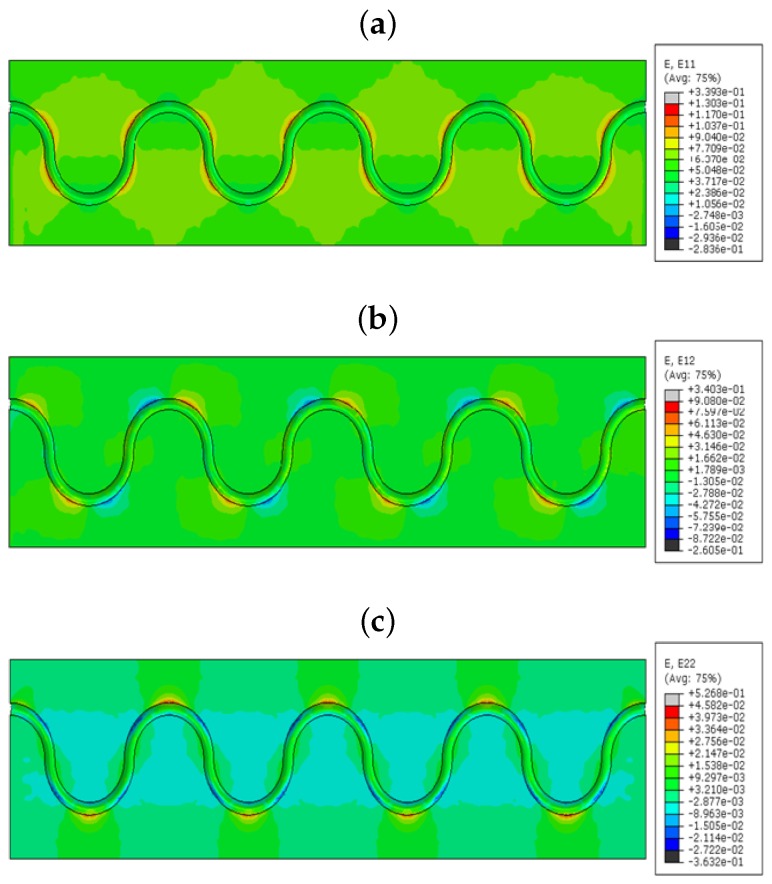
(**a**) $E_{11}$ strain component; (**b**) $E_{12}$ strain component; (**c**) $E_{22}$ strain component (polyimide-coated FOS numerical model; the value observed in the host epoxy), sinusoidal alignment.

Figure 10b shows the strain in the shear direction of the numerical model. In this case, there is a spot of compression in the peak. From this, we can observe the strain measurement of the transverse direction, also. The maximum strain shows 0.4395, which is quite high compared to the longitudinal tensile strain. The minimum also goes to 0.3416, which has a lower compression strain than the tensile one. One side of the peak of the fiber feels the tensile strain, and the opposite side feels compression strain in the peak [11]. Figure 10c describes the strain effects over the lateral axis of the model. In this finite element model, we get more compression strain concentration over the curvatures of the sinusoidal shape rather than the tension strain of 0.6794 noted in the peak of each curvature. The FOS coating is modeled with polyimide coating and submitted for the simulation with the tensile loading condition as the previous models. Figure 11a shows linear, lateral and shear strains, respectively, for this model. It displays the path of the optical fiber, which has some strain influence in the bending edges of the curvature. The maximum strain on the part is 0.339, and the minimum is 0.3805 mm/mm. Figure 11b corresponds to the shear strain of embedded optical fiber with polyimide coating inside the green epoxy zone. It shows shear strain effects on the polyimide coating material, where the higher strain is 0.3403. It has the lowest strain of 0.2605, which is more compressed than the longitudinal configuration. In total, it seems that the maximum shear strain in this model is less than the maximum shear strain in the acrylate model. Similarly, the lowest strain is also less than the acrylate model. However, both models are able to identify the tensile and compression strains with different values. Figure 11c shows the lateral configuration with the maximum strain value of 0.5268. This value is lower compared to the model with acrylate coating.

**Figure 12:**
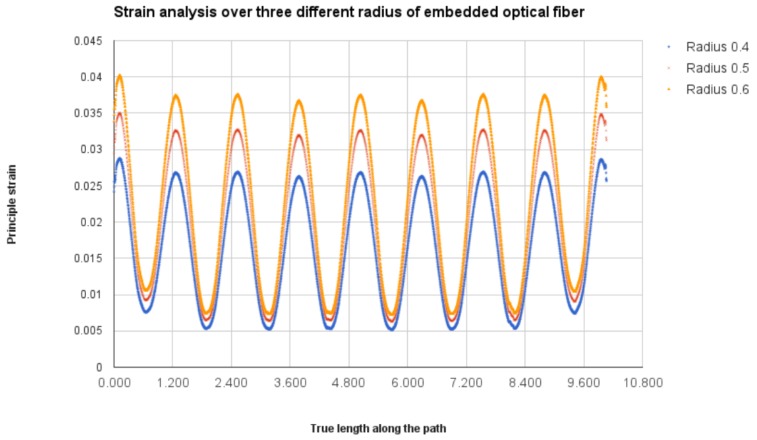
Principal strain variation based on sinusoidal peak variation.

The fiber alignment parameter is modified to check the sensor proximity to the edge of the specimen length. Therefore, the radius of the sinusoidal peak (R) in Figure 3 is varied 0.4, 0.5 and 0.6 mm to check if the strain value is more than the maximum strain value of epoxy. From Figure 12, we notice that the strain value in the 0.6-mm radius model reaches 4% strain, which is close to the maximum strain of the epoxy material [29]. This may lead to the fiber slippage or material property modification to the host material. If the sinusoidal peaks are further positioned close to the edge, the maximum strain values will go to a substantial level that would affect the sensor and material properties. For the other two models, the strain values stay inside the limit. Therefore, the fiber provides some clearance to the edge in order to reduce the edge effect in the monitoring process.

### 5.2. Results of Experimentation

**Figure 13:**
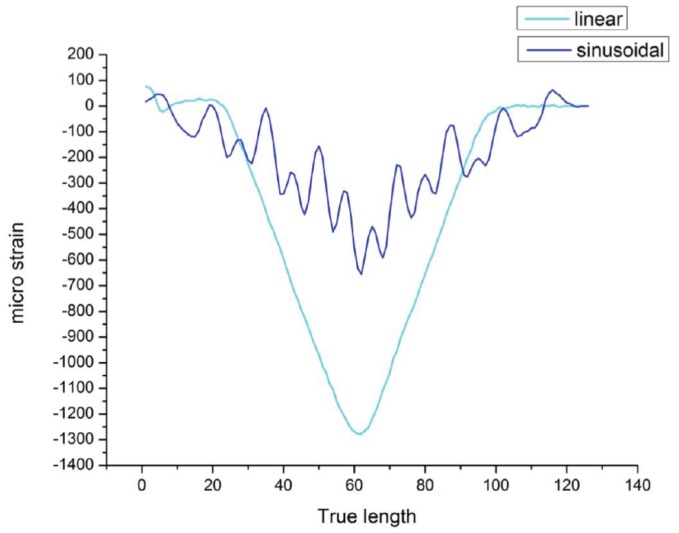
Linear vs. sinusoidal alignment, bending load.

**Figure 14:**
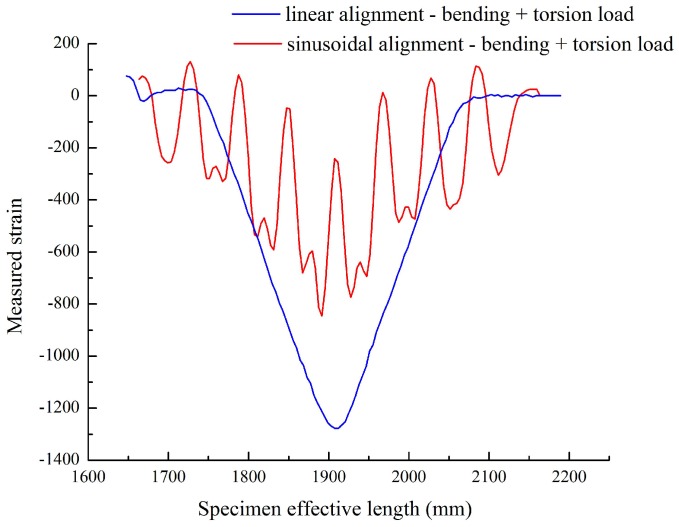
Linear vs. sinusoidal alignment, bending + torsional load.

**Figure 15:**
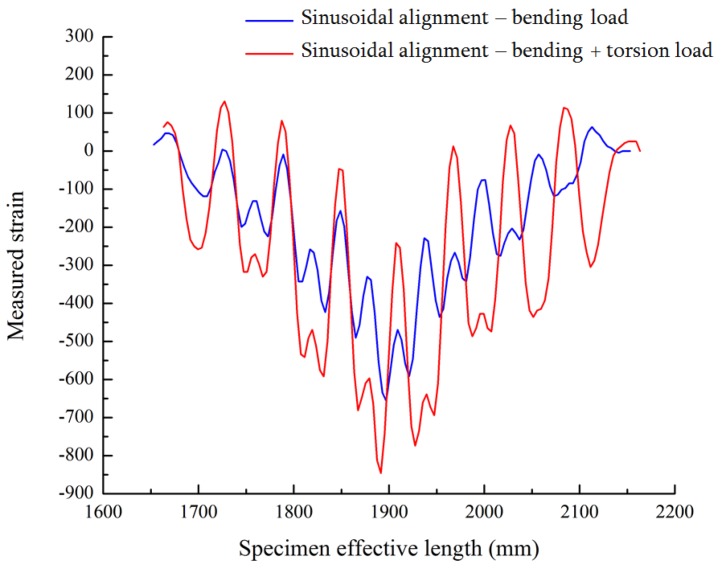
Sinusoidal alignment, bending Load with torsion vs. without torsion.

The distributed FOS alignments are studied to see the effectiveness of sensor positioning for their sensitivity, the elastic range of measurements and multi-parameter measurements (strain at bending and torsion). It is assumed that linear alignment sensors are able to observe lateral stresses, but not sensible on longitudinal and shear loads (see Figure 8) from the numerical simulation. Therefore, sinusoidal positioning is considered as a better solution for the various directions of strain sensing and also for the large surface area coverage from a single FOS. Figure 13 shows the linear and small sinusoidal alignment FOS behavior under bending load. Both methods would provide the strain detail, but the range of the strain value observed by the small sinusoidal model is half the range of the strain value observed from the linear alignment. Furthermore, Figure 14 shows that the sinusoidal alignments are more sensible for the torsion load applied. There is no difference in the linear configuration between torsion and torsion + bending load; both provide the same strain value of 1300 μm/m. However, in the sinusoidal fibers, the curve clearly shows the peaks; these peaks’ strain values show the effect of torsion. Figure 15 shows the comparison of peaks from the effect of torsion. Without the torsion load, the peak value reaches 100 μm/m, but with torsion load, the peak value reaches the strain value of 250 μm/m; this value represents the torsion load applied at the edge of the specimen.

**Figure 16:**
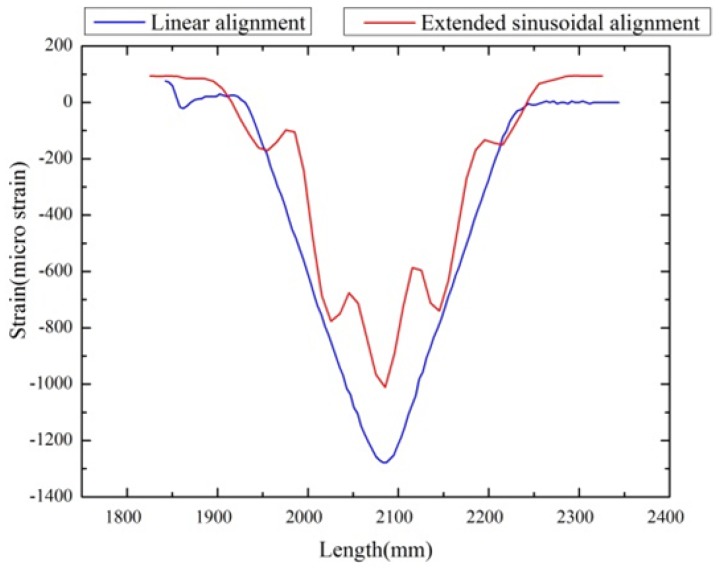
Linear vs. extended sinusoidal alignment, bending load.

**Figure 17:**
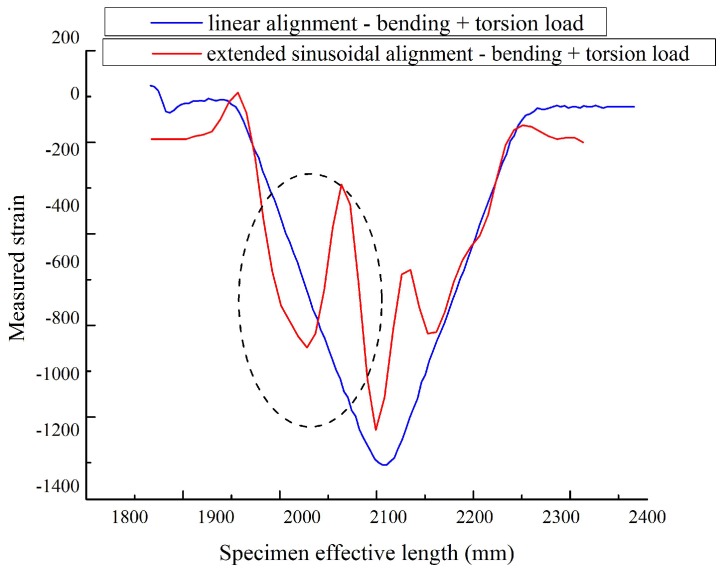
Extended sinusoidal alignment, bending load with torsion vs. without torsion; the circled highlighted area indicates the torsion effect.

However, the variation between the linear and sinusoidal aligned FOS strain values is still high; it is around 600 μm/m for the bending load. Additionally, the variation is 400 μm/m for bending + torsion load (see Figure 14). To recover this strain value, the sensors are thought to extend with an enlarged period, by assuming that the extended fiber could provide the strain value close to the linear alignment configuration. Therefore, the extended sinusoidal alignment was tested under bending and torsion load, and their strain values are compared with the linear model. The extended sinusoidal aligned FOS provides the strain value equal to 1050 μm/m (see Figure 16), which is closer to the strain observed in the linear configuration of 1300 μm/m. In addition, the torsion load provides a clear difference in the peaks of the sinusoidal curve with observable variation (see Figure 17).

## 6. Discussion

The comparison between the linear and sinusoidal configuration is shown in the figures from the numerical simulation and experiments. Additionally, these figures compare fiber optic strain sensing properties. The strain observed in the epoxy is assumed as the strain seen by the fiber optics; because the host material provides the external stress to the FOS structure to modify the wavelength or the travel time of the waves. This modification is converted to the strain value during the signal treatment of FOSs. Therefore, this study can clearly present the FOS’s sensing capability according to the host material. The sinusoidal and linear alignment should have provided different intrusive properties, which would lead to the modification in the mechanical property of the material. However, this intrusive characteristic is more effective while embedded between composite plies. In our case, we insert the sensor in the bonding material. Therefore, the material property variation produced by this insertion characteristic is negligible.

As we bond the sensor over a structure, the host composite material became unimportant for this study; however, the consideration of the composite material provides the orthotropic condition to check the approximate behavior of the real condition. Therefore, the carbon fiber-reinforced composite material is considered for the numerical study and glass fiber-reinforced composite for the experimental method. The composite materials’ variation affects the strain value comparison between the numerical and experimental method, but we are interested in the strain component rather than the strain values during this comparison. The strain values are compared while the same materials are in use (comparison within the numerical model and comparison within the experiment). In addition, the future detailed study of the strain value comparison would help to create the analytic function about alignment.

In the linear numerical model, the sensor is not valuable with respect to collecting linear and shear strains, because the tensile load does not provide enough stress to the sensor. It acts to extend the composite and epoxy towards the longitudinal direction. In the same way, shear load does not make any section variation to provide the $\gamma_{xy}$ strain in the normal direction, which is along the lengthwise direction. Therefore, no visible high valued shear strain is observed around the fiber optic zone. However, epoxy has a high Poisson ratio that provides a high lateral strain. This lateral strain transformed to the FOS, which is embedded in epoxy. Therefore, the lateral strain is clearly visible in the linear FOS alignment configuration. The coating material acrylate and polyimide do not change this functionality, but they can vary the strain values by changing the transfer of stress between the FOS and the epoxy, as coatings have different modulus and material properties. The FOS with acrylate coating provides more strain values in all directions compared to the FOS with polyimide coating. This proves the advantage of polyimide coating over acrylate; because the high strain value in epoxy may lead to damage in the composite material assembly. On the other hand, the strain values are observed with minor variation between acrylate and polyimide coating. This should be compared with the cost of the FOS based on the coating material. The polyimide-coated FOS costs 15% more than the one with acrylate coating [2].

The coating difference creates less variation of the strain value observed in the numerical simulation. Therefore, the users should take into account this factor based on the application. If the application is large scale with atmospheric temperature, the small strain modification will not play a big role in material damage. Therefore, the acrylate-coated FOS is preferable. On the contrary, for a micro-scale study or a high temperature-based application, this small variation makes a big difference, so the polyimide-coated sensor is preferable.

From the strain values in the numerical model sinusoidal configuration, we observed that the $E_{11}$ took place by the tensile strain component around the curvature of the sinusoidal alignment (reddish color, Figure 10a); shear strain $E_{12}$ came off with the combination of compression and tensile combined strains (red and blue, Figure 10). $E_{12}$ provides lateral tensile strains (reddish, Figure 10) and a small amount of compression strain in the curvatures (bluish, Figure 10). The value of the strain varies based on the coating material used in the FOSs. This proves that the sinusoidal FOS alignment is better for the multi-axial sensing for a large structure. Some structures expect specified axis strain sensing rather than single axis strain. For those cases, the linear alignment FOS might not be effective. Therefore, the multi-parameter strain sensing characteristic sinusoidal alignment is preferable.

Once the directional strain is proven, it is important to analyze the strain values during the monitoring of the structure; because sensors strain values should reflect clearly the amount of anomalies to identify the risk in the structure. The sinusoidal alignment can reduce the strain range or value when embedded in the structure. While comparing linear alignment with small sinusoidal alignment FOS, the strain value is noticed to be exactly half from 1300 μm/m to 600 μm/m. However, we are able to detect the strain by peaks under torsional load by sinusoidal alignment. In Figure 15, strain by bending load values jumps from 100 μm/m to 250 μm/m while applying the additional torsion load. This torsional effect is observed in the peaks mentioned in Figure 15. The torsional strain can represent the shear strain observed in the numerical simulation part, and it works also under bending load.

Our objective is to reach the strain value close to the linear alignment configuration by keeping the torsional load strain sensing. Therefore, the extended sinusoidal FOS alignment is considered, and they are tested under the same boundary condition to check the strain by the optical back scatter method. The strain value from this novelty positioning method (1050 μm/m) approaches the strain value of the linear positioning (1300 μm/m) method for a given boundary condition. This proves that it is possible to recover the strain value range with the multi-parameter sensing. Additionally, torsional strain is also observed with peak variation (see Figure 17). This provides an outlook that the large-scale composite structure SHM is possible with multi-directional strain sensing by using our proposed fiber optical sinusoidal alignment procedure.

## 7. Conclusions

FOSs are preferable for the SHM of large structures like wind turbine blades. However, they have a difficulty in identifying multi-axial strains while embedded in the structure. It is also hard to distinguish the strain coordinates, if the loading direction is random. In this study, we have discussed the optimal solution for multi-axial strain sensing by working on distributed FOS alignment in fiber-reinforced composite with epoxy bonding.

From our numerical study, the multi-parameter strain is clearly visible in the sinusoidal alignment method compared to the linear alignment method. Linear alignment FOS shows only lateral strains, while the sinusoidally-aligned FOS provides the strain information in the linear, shear and lateral directions. This strain value varies based on the coating material considered. Acrylate coating permits high strain to the host material compared to the polyimide coating.

The multi-axial strain sensing of FOS was also proven by the experiment method under bending and torsion loads. The sinusoidal aligned FOSs are able to measure the strain results by bending and torsion load. In contrast, linear alignment FOS only detects the strain components of bending load. However, the strain value (1300 μm/m) collected by linear (or straight) alignment FOS was higher than the one of sinusoidal alignment FOS (600 μm/m), for similar boundary conditions. The linear alignment’s strain range was 55% higher than the simple sinusoidal configuration. To approach the strain range towards the linear alignment FOS’s strain, the periods of sinusoidal alignment were extended. This extended sinusoidal alignment FOS helps to come closer to the strain magnitude (1050 μm/m) observed in linear alignment FOS. The difference between linear and sinusoidal configuration’s strain values was reduced to 20%. This study proves that it is possible to have a multi-parameter strain sensing in distributed FOS without affecting the strain magnitude. In addition, it gives the overview to the users for making an optimal SHM system for large-scale structures and bonding.

## Figures and Tables

**Table 1:** Mechanical properties of the materials used in this study; source: [24,26].

Materials	Density (kg/m${}^{3}$)	Modulus (MPa)	Poisson’s Ratio
Carbon composite (CFRP)	1950	$E_{1}$ = 103,000, $E_{2}$ = 10,400, $G_{12}$ = 54,000	$\nu_{12}$ = 0.3, $\nu_{21}$ = 0.03
Epoxy	1250	3500	0.3
Acrylate	950	2700	0.35
Polyimide	1100	3000	0.42
Silica glass	2400	72,000	0.17
